# The Development of a Fluorescent Microsatellite Marker Assay for the Pitaya Canker Pathogen (*Neoscytalidium dimidiatum*)

**DOI:** 10.3390/genes15070885

**Published:** 2024-07-05

**Authors:** Rui Li, Xi Li, Jingcheng Tang, Changping Xie, Jianan Wang

**Affiliations:** 1 School of Tropical Agriculture and Forestry, Hainan University, Haikou 570228, China; 20095132210102@hainanu.edu.cn (R.L.); lixi1792@163.com (X.L.); 2021220951320087@hainanu.edu.cn (J.T.); xiechangping009@163.com (C.X.); 2 Key Laboratory of Green Prevention and Control of Tropical Plant Diseases and Pests, Ministry of Education of China, Haikou 570228, China

**Keywords:** *Neoscytalidium dimidiatum*, draft genome, fluorescent, SSRs, population genetics

## Abstract

Pitaya canker, caused by *Neoscytalidium dimidiatum*, is a destructive disease that significantly threatens the safety of the pitaya industry. The authors of previous studies have mainly focused on its biological characteristics and chemical control. However, there are no molecular markers available thus far that can be used for the population genetics study of this pathogen. In the present study, a draft genome of *N. dimidiatum* with a total length of 41.46 MB was assembled in which 9863 coding genes were predicted and annotated. In particular, the microsatellite sequences in the draft genome were investigated. To improve the successful screening rate of potentially polymorphic microsatellite makers, another five *N. dimidiatum* isolates were resequenced and assembled. A total of eight pairs of polymorphic microsatellite primers were screened out based on the polymorphic microsatellite loci after investigating the sequencing and resequencing assemblies of the six isolates. A total of thirteen representative isolates sampled from different pitaya plantations were genotyped in order to validate the polymorphism of the resulting eight markers. The results indicated that these markers were able to distinguish the isolates well. Lastly, a neighbor-joining tree of 35 isolates, sampled from different pitaya plantations located in different regions, was constructed according to the genotypes of the eight molecular markers. The developed tree indicated that these molecular markers had sufficient genotyping capabilities for our test panel of isolates. In summary, we developed a set of polymorphic microsatellite markers in the following study that can effectively genotype and distinguish *N. dimidiatum* isolates and be utilized in the population genetics study of *N. dimidiatum*.

## 1. Introduction

Pitayas (*Hylocereus* spp.: Cactaceae), also known as kylin fruit, valentine fruit, and red dragon fruit, are native to tropical areas of North, Central, and South America [1] and are widely cultivated in many tropical and subtropical countries owing to their high nutritional, medicinal, and ornamental value [2,3]. As of December 2020, data from China’s Ministry of Commerce indicate that the planting area of pitayas across the country covers approximately 35,555 hectares [4]. In 2021, the planting area of pitayas in China exceeded 66,667 hectares, and the fruit’s yield exceeded 1.6 million tons, with China becoming the top country worldwide for production [5]. The main planting areas in China include the provinces of Hainan, Guangdong, Guizhou, and Yunnan and the Guangxi autonomous region [6].

*Neoscytalidium dimidiatum*, the causal agent of pitaya stem canker, can significantly affect pitaya production. The authors of a recently published comprehensive review proposed that *N. dimidiatum* is synonymous with *N. hylocereum*, *Scytalidium hyalinum*, *N. novaehollandiae*, *N. orchidacaearum*, etc. [7]. The stem canker of pitaya not only harms plant growth and the quality of fruits but also causes huge economic losses. For example, canker disease became the most significant disease of pitayas in southern Florida in 2015, and symptoms of the disease were particularly evident in fruits, with an incidence of up to 70% [8]. A survey on pitaya diseases in 2014 in Qionghai City of the Hainan Province indicated that the incidence rate of pitaya canker and stem rot reached 100% [9]. To date, there have only been a few studies on the biological characteristics and chemical control of *N. dimidiatum* [6,10]. From an evolutionary perspective, the population structure of pathogen populations characterizes its evolutionary potential, and high genetic diversity ensures survival and provides strong evolutionary advantages [11]. However, the distinct lack of powerful molecular markers has limited the population genetics studies and risk assessment of this pathogenic population.

Next-generation sequencing (NGS), also known as high-throughput sequencing (HTS), has made it possible to perform deep, high-throughput, and high-quality sequencing of a species of interest and the construction of a draft genome sequence, facilitating our understanding of the derived genetic information from a genomics perspective [12]. In addition, the use of NGS technology has made it possible to leverage a variety of molecular markers in genetics and genomics studies, such as microsatellite markers and single-nucleotide polymorphism (SNP) markers, among others.

A microsatellite sequence (MS), also known as simple sequence repeats (SSRs) or short tandem repeats (STRs) [13], is generated by sliding mismatch during DNA replication or repair or unequal exchange during division [14]. It is generally believed that microsatellites are subject to neutral selection [15]. Because of high mutation rates and neutral selection, a high number of microsatellite alleles may accumulate within the species genome [16]. Thus, microsatellite markers have been widely used as valuable genetic markers; however, genome-wide SNP markers have become popular for use in population genetics studies [17,18]. Using these microsatellite genotype data, we can not only understand the changes in pathogen population structure and genetic diversity but also understand the origin center, transmission route, inheritance migration, or other evolutionary mechanisms of pathogens because microsatellite genotypes are not or are rarely influenced by natural selection during the evolutionary process [11,19].

In the following study, we aimed to use NGS technology to assemble and annotate the draft genome of *N. dimidiatum* and perform whole-genome resequencing of another five representative isolates of *N. dimidiatum* collected from different locations in China. The polymorphic microsatellite loci between the draft genome sequences and the other five resequencing assemblies were mined. Finally, a total of eight polymorphic microsatellite markers were screened after examining a set of thirteen representative isolates with broad geographic and genetic backgrounds.

## 2. Materials and Methods

### 2.1. Sampling and Isolation

The *N. dimidiatum* isolate HNDZ1920 used for draft genome sequencing was sampled from the stems of a pitaya plant in Danzhou City, Hainan Province, in August 2019. With regard to the other five isolates used for resequencing, three isolates were originally isolated from the diseased and healthy junctions of pitaya stems with typical symptoms of canker collected in Ledong County, Hainan Province; one isolate was isolated from Sanya City, Hainan Province; and the fifth isolate was isolated from Yangjiang City, Guangdong Province (Table 1). The above isolates were recovered from symptomatic tissues taken from diseased plants; samples (0.5 × 0.5 cm) from lesion margins were surface disinfected for 10 min with 0.3% NaClO, plated on potato dextrose agar (PDA), and incubated at 26 °C for five days. All isolates were purified using the single-spore technique, and all of the purified isolates were transferred to and maintained on PDA medium at 28 °C.

### 2.2. DNA Extraction

The purified isolates were grown on a PDA medium at 28 °C for five days prior to DNA extraction. The mycelium was scraped and ground with liquid nitrogen. The whole-genome DNA was extracted using a Super Plant Genomic DNA Kit (TIANGEN, Cat. No. 4992879), detected via 1% agarose gel electrophoresis, qualified using a Nanodrop microspectrophotometer, and ultimately stored at −20 °C in a refrigerator.

### 2.3. Isolate Identification

All isolates were determined using morphological and molecular biological methods. Regarding the morphological identification method used, the isolates were inoculated onto the PDA culture plates and incubated for 7 d at 28 °C in an incubator, and the morphology, color, and texture of the colonies were observed and recorded. In particular, the conidia, arthrospores, and chlamydospores were used as the morphological basis for the identification of the pathogens.

After morphological determination, the molecular biology identification approach was utilized to identify the sampled isolates. Specifically, the rDNA ITS and *β-tub* region of the *N. dimidiatum* genome were amplified with universal primer pairs ITS1/ITS4 [20] and Bt2a/Bt2b [21]. The primer sequences were as follows: ITS1: 5′-TCCGTAGGAGAACCTGCGG-3′; ITS4: 5′-TCCTCCGCTTATTGATATGC-3′; Bt2a: 5′-GGTAACCAAATCGGTGCTGCTTTC-3′; and Bt2b: 5′-ACCCTCAGTGTAGTGACCCTTGGC-3′. After PCR applications, 3 μL of the PCR products was detected using 1% agarose gel electrophoresis. Next, the PCR products were Sanger sequenced by Sangon Biotech (Shanghai) Co., Ltd. Lastly, a total of 41 sequences belonging to the Botryosphaericeae family were downloaded from the GenBank database (Table 2), and PhyloSuite v1.2.3 [22,23] software was used to construct a phylogenetic tree using the maximum likelihood (ML) method based on the combined ITS and *β-tub* sequence alignment. *Botryosphaeria dothidea* (CMW 8000) was set as an outgroup. Both of the nucleotide substitution models of the two loci were inferred as HKY+F, and the number of bootstraps [24,25] was set as 5000.

### 2.4. Genome Assembly and Annotation

To determine whether the extracted genomic DNA of isolate HNDZ1920 met the requirements for whole-genome sequencing, concentration, purity, and integrity tests were conducted using an Agilent 5400 (Advanced Analytical Technologies, Santa Clara, CA, USA). Next, the genomic library was prepared using the NEBNext^®^Ultra™ DNA Library Prep Kit for Illumina (NEB, San Diego, CA, USA). Genome sequencing was performed with the Illumina NovaSeq PE150 platform by Novogene Co., Ltd (Beijing, China). Afterward, the draft genome of HNDZ192 was assembled and annotated. Specifically, we used FastQC (https://www.bioinformatics.babraham.ac.uk/projects/fastqc/, accessed on 31 October 2021), FastUniq [26], Musket [27], and Fastp [28] for quality control. Next, we used GCE [29] and JellyFish [30] for the genome survey. Furthermore, we used kmergenie (http://kmergenie.bx.psu.edu/, accessed on 31 October 2021), SPAdes [31], ABySS [32], SOAPdenovo2 [33], quickmerge [34], and gapcloser [33] for genome assembly. Lastly, we used QUAST [35] and BUSCO [36] to evaluate the genome assembly.

The genome components were annotated via CEGMA [37], Augustus [38], RepeatMasker [39], RepeatModeler [40], tRNAscan [41], barrnap (https://github.com/seemann/barrnap, accessed on 14 September 2022), and CMscan 1.1.4 [42] software and the Rfam [43] database. Additionally, the genome functions were annotated via commonly used constructed databases locally with Blastp [44], including Nr [45], Kog [46,47], KEGG [48,49], GO [50], SwissProt [51], Pfam [52], TCDB [53], P450 [54], and Carbohydrate-Active enZYmes (CAZymes) [55]. To explore the gene clusters involved in the formation of secondary metabolites (SMs) within the genome, antiSMASH [56] was used. Moreover, we used the PHI [57] and DFVF [58] databases to annotate the pathogen–host interactions and fungal virulence factors.

The traditional development strategy of microsatellite markers requires the synthesis of a large number of primers and amplifications of each sample in the screening population, resulting in the wasted synthesis of a large number of monomorphic primers [59]. In order to improve the screening efficiency of polymorphism microsatellite loci, we integrated our technique with resequencing technology to screen the polymorphic variations of microsatellite loci among different isolates of *N. dimidiatum*. The whole-genome resequencing of the other five isolates was performed using the Illumina NovaSeq PE150 platform by Novogene Co., Ltd., with a sequencing depth of 40×. The resequencing genome assemblies were processed the same as the draft genome of HNDZ1920.

### 2.5. Microsatellite Marker Development and Primer Design

The microsatellite markers used in the present study were developed according to the following two strategies. At the start of the study, we utilized MISA v2.1 [60] software to search and identify SSR loci on the assembled HNDZ1920 draft genome sequence only, and the parameters for “unit_size” were set as follows: 1, 2, 3, 4, 5, and 6; the corresponding parameter “min_repeats” was set as 10, 6, 5, 5, 5, and 5, respectively. The compound SSR loci were defined if the maximum number of bases between two distinct SSRs was 100. We ruled out the single-base and compound SSR loci and ran MISA to screen the remaining SSR loci for primer design. In order to identify extra SSR loci, we further aligned these remaining SSRs and their flanking sequences with the resequencing assemblies of another five isolates. All SSR loci with genetic variations in a particular locus among different assemblies were extracted. These candidate SSR loci were used to design the additional primers using Primer3 v2.3.6 [61]. The program parameters were set as follows: the range of the fragment lengths of amplicons was from 100 to 350 bp; the positions of the amplicon fragments were from the first base to the last five bases of the repeat sequence; and the remaining parameters were defaulted.

To examine the polymorphic primers, a total of thirteen *N. dimidiatum* isolates with diverse genetic backgrounds were amplified using the PCR method. The designed primers were synthesized using the splicing method and a 21 bp adapter sequence was added to the upstream primer during synthesis. The reaction was performed using a Veriti 384 PCR instrument and the PCR amplification program was set as follows: initial denaturation (95 °C, 5 min); denaturation (95 °C, 30 s), gradient annealing (62–52 °C, 30 s, and decreasing 1 °C per cycle), and extension (72 °C, 30 s), 10 cycles; denaturation (95 °C, 30 s), annealing (52 °C, 30 s), and extension (72 °C, 30 s), 25 cycles; extension (72 °C, 20 min); and storage (4 °C). The total 10 μL PCR mixture was composed of 5 μL of 2×Taq PCR Master Mix, 1 μL of template, 0.5 μL of forward primer (10 pmol/μL), 0.5 μL of reverse primer (10 pmol/μL), and 4 μL of distilled water. Following PCR amplification, we detected the products using an ABI 3730xl fluorescence-based DNA analyzer; the results were analyzed using GeneMarker v3.0.0 [62] software, allowing us to determine the number of alleles, peak charts, and genotype data.

We added four different fluorophores, including 6-FAM [5′-fluorescein ce-phosphoramidite], 6-HEX [5′-hexachlorofluorescein phosphoramidite], 6-ROX [6-carboxy-x-rhodamine], and 6-TAMR-SE [6-carboxytetramethylrhodamine N-succinimidyl ester], to the 5′ end of the polymorphism PCR primers. To validate the power of these fluorescent microsatellite markers, 35 isolates collected from different locations in China were genotyped using the resulting eight pairs of polymorphic microsatellite primers and detected via ABI 3730xl fluorescence-based capillary electrophoresis. Lastly, we used the genotyping data to construct a neighbor-joining tree through the use of the aboot() method in the R package poppr [63]. The number of bootstrap replicates was 1000.

## 3. Results

### 3.1. Isolate Identification

**Figure 1:**
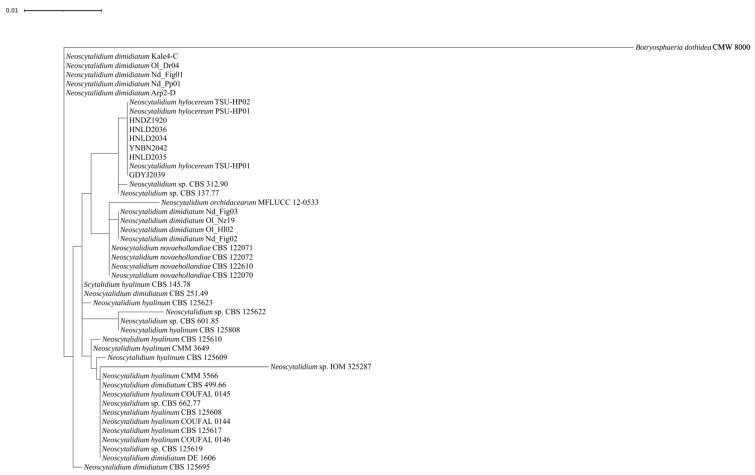
Phylogenetic tree of *Neoscytalidium* based on the combined ITS and *β-tub* sequence alignment. The tree was rooted to *B. dothidea*. Note that the figure only shows the six *N. dimidiatum* isolates used during genome sequencing and resequencing.

To identify all the isolates used in the present study, a phylogenetic tree was constructed based on the alignments of ITS and *β-tub* loci. The result showed that all isolates were clustered with *N. dimidiatum* isolated from the stem cankers of pitayas, indicating that all isolates used in the present study were *N. dimidiatum* (Figure 1).

### 3.2. Genome Assembly and Annotation

**Figure 2:**
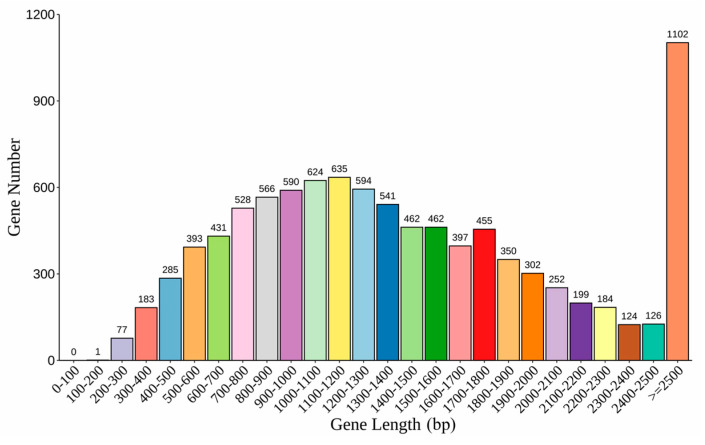
Gene length distribution.

The resulting genome assembly harbored a total genome size of 41.46 MB with an overall GC content of 53.96%. The genome sequence was assembled with a contig N50 length of 266.9 KB. The quality of the genome assembly was evaluated using the Benchmarking Universal Single-Copy Orthologs (BUSCO) tool with the “fungi_odb10” library as the reference dataset. The results contained 729 groups, accounting for 96.2% of a total of 758 complement ortholog groups in BUSCO. Approximately 6.45% of the repeat sequences were identified via RepeatModeler and RepeatMasker. The content of tRNA, rRNA, sRNA, and snRNA was identified as 138, 15, 3, and 38, respectively. The number of protein-coding genes annotated using CEGMA and AUGUSTUS was 9863 (Table 3). The lengths of the genes were widely distributed (Figure 2).

**Figure 3:**
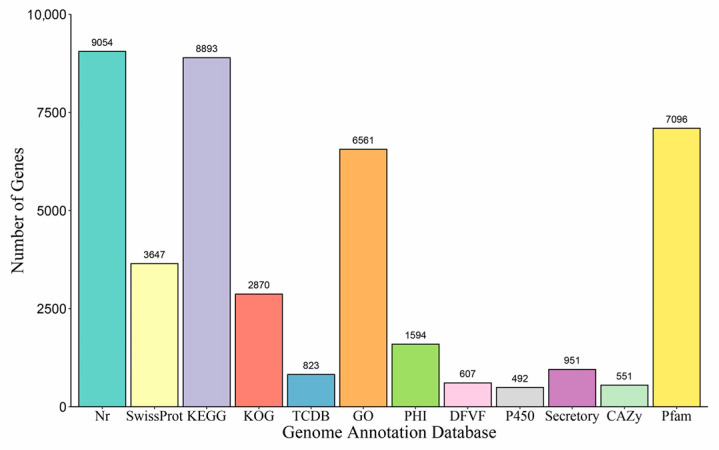
The number of genes annotated using coding genes in the different databases.

**Figure A1:**
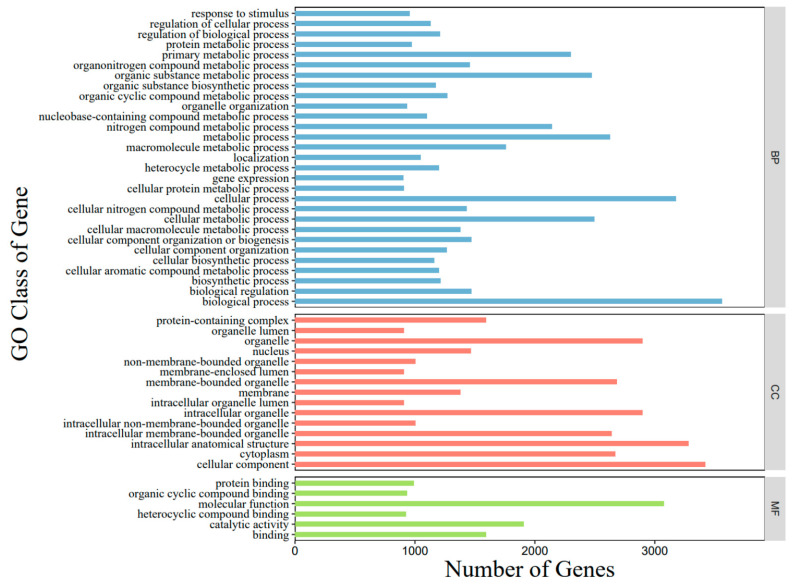
Gene Ontology (GO) annotation of the *N. dimidiatum* draft genome.

**Figure A2:**
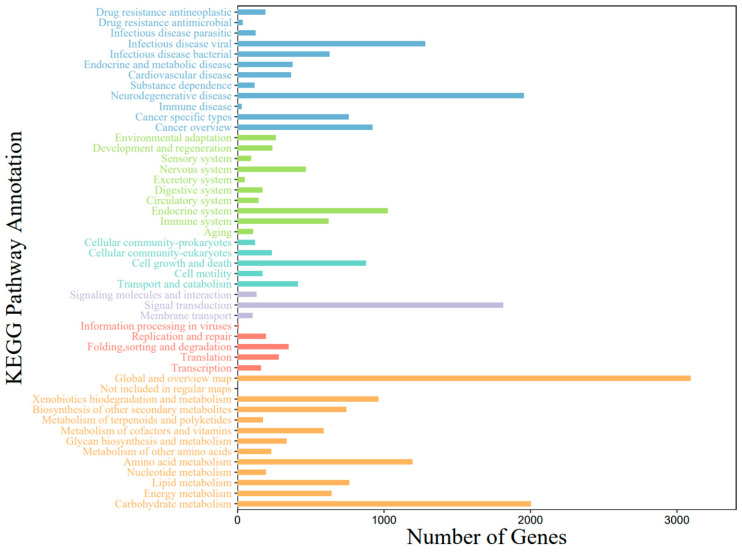
Kyoto Encyclopedia of Genes and Genomes (KEGG) pathway annotation of the *N. dimidiatum* draft genome.

**Figure A3:**
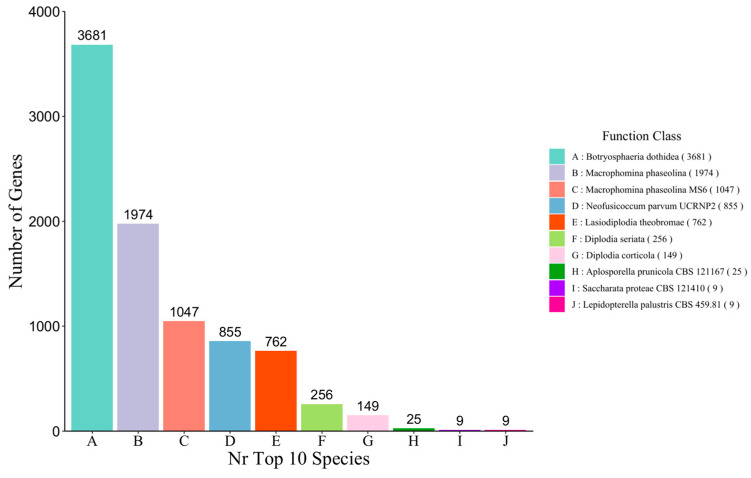
RefSeq non-redundant protein annotation result of the *N. dimidiatum* draft genome.

**Figure A4:**
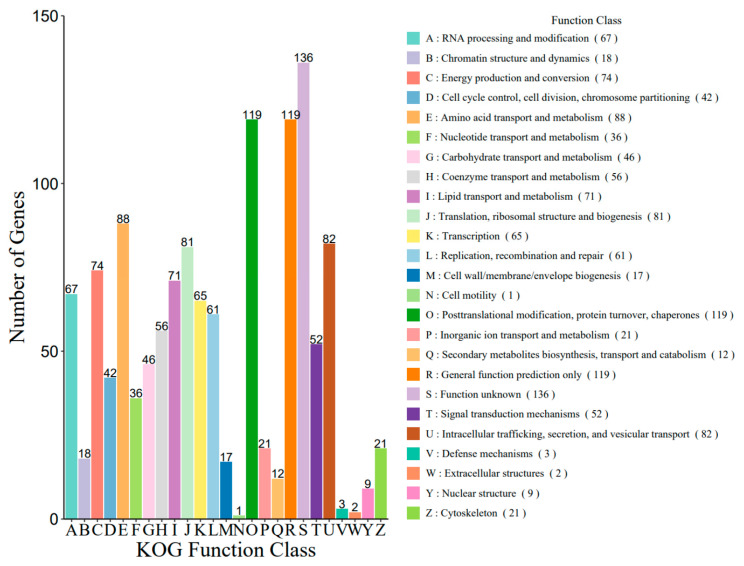
Clusters of orthologous groups for the eukaryotic complete genome annotation of the *N. dimidiatum* draft genome.

**Figure A5:**
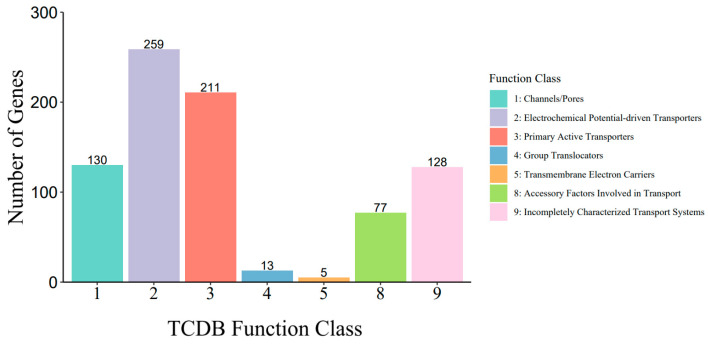
The Transporter Classification Database annotation of the *N. dimidiatum* draft genome.

**Figure A6:**
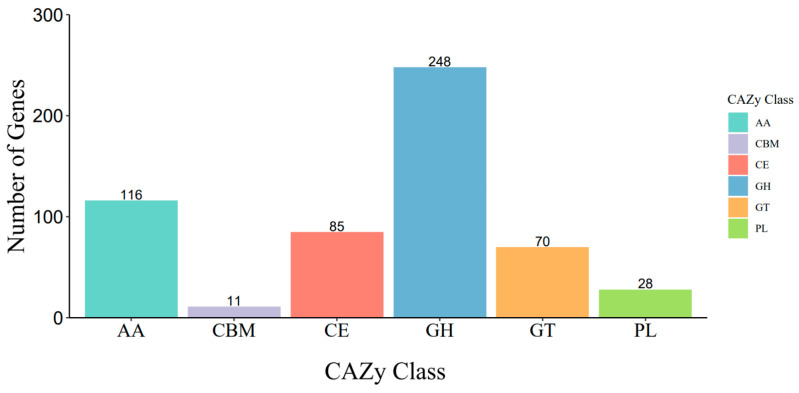
Carbohydrate-Active enZYmes Database annotation of the *N. dimidiatum* draft genome.

**Figure A7:**
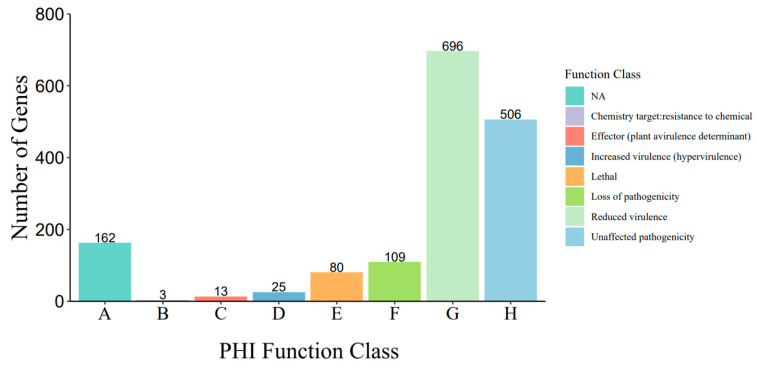
Pathogen–host interactions base annotation of the *N. dimidiatum* draft genome.

**Figure A8:**
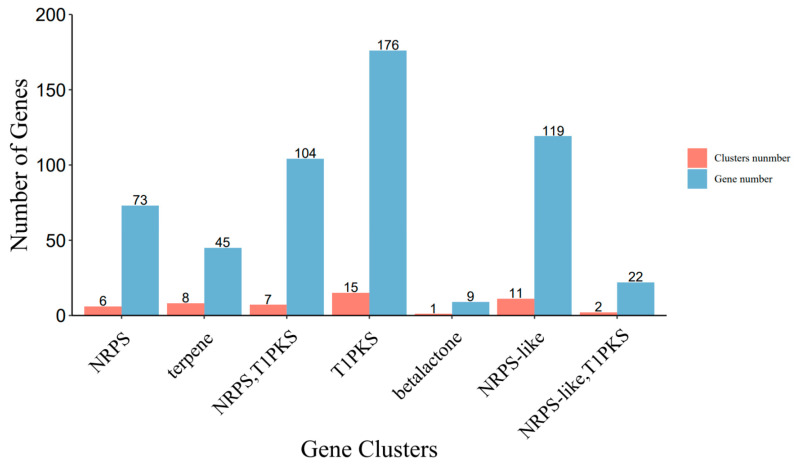
Secondary metabolic cluster analysis of the *N. dimidiatum* draft genome.

A total of 9054 genes were annotated using the NR Fungi sub-database; 6561 genes were annotated with the GO database; 2870 genes were annotated with the KOG database; and 3749 genes were annotated with the SwissProt database. In addition, 8893 and 7096 genes were assigned to KEGG and Pfam terms, respectively. A total of 951 genes were defined as encoded secreted proteins using SignalP and TMHMM, respectively. A total of 551 genes were identified using dbCAN2, including 116 auxiliary activities (AAs), 248 glycoside hydrolases (GHs), 85 carbohydrate esterases (CEs), 70 glycosyl transferases (GTs), and 28 polysaccharide lyases (PLs). In addition, 823 and 492 genes were annotated according to the TCDB database and Cytochrome P450 database, respectively. Ultimately, fifteen type I polyketides (T1PKS), six non-ribosomal peptide synthetases (NRPS), eleven non-ribosomal peptide synthetases (NRPS), and eight terpene gene clusters were identified. Lastly, a total of 1594 PHI entries and 607 DFVF entries were annotated (Figure 3 and Figure A1, Figure A2, Figure A3, Figure A4, Figure A5, Figure A6, Figure A7 and Figure A8). Similarly, after whole-genome resequencing assembly, we obtained five genome sequences with lengths of 42.64 MB, 42.84 MB, 43.03 MB, 42.95 MB, and 43.11 MB.

### 3.3. Polymorphic Microsatellite Markers

**Figure 4:**
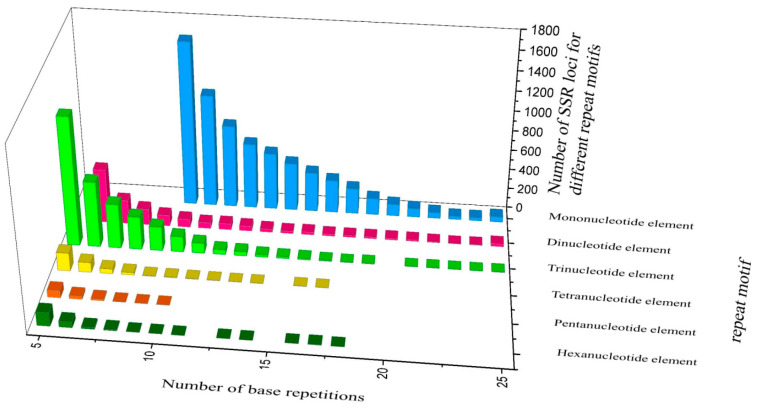
The number of genomic microsatellite loci in the *N. dimidiatum* genome.

A total of 12,515 microsatellite loci were identified in the reference genome sequence, and the main types of repeat motifs were mononucleotide (A/T and C/G) and trinucleotide (CCG/CGG) (Figure 4).

Based on the reference genome sequence only, 2865 pairs of microsatellite primers were screened, and 192 pairs of primers were randomly selected and synthesized by Guangzhou Tianyihuiyuan Gene Technology Co., Ltd. The resulting primer pairs were tested via the screening panel, including thirteen *N. dimidiatum* isolates, and two pairs of polymorphic primers were screened successfully.

Combined with the alignments of the five resequencing assemblies to the draft genome, we successfully designed 3233 pairs of primers on the loci, thus ruling out single-base and composite SSR loci. We preliminarily selected 336 pairs of primers randomly and screened them using the thirteen isolates. A total of six pairs of primers that can amplify more than two alleles were screened, according to the peak output condition and quality. Taken together, a total of eight pairs of polymorphic primers were developed successfully.

**Figure 5:**
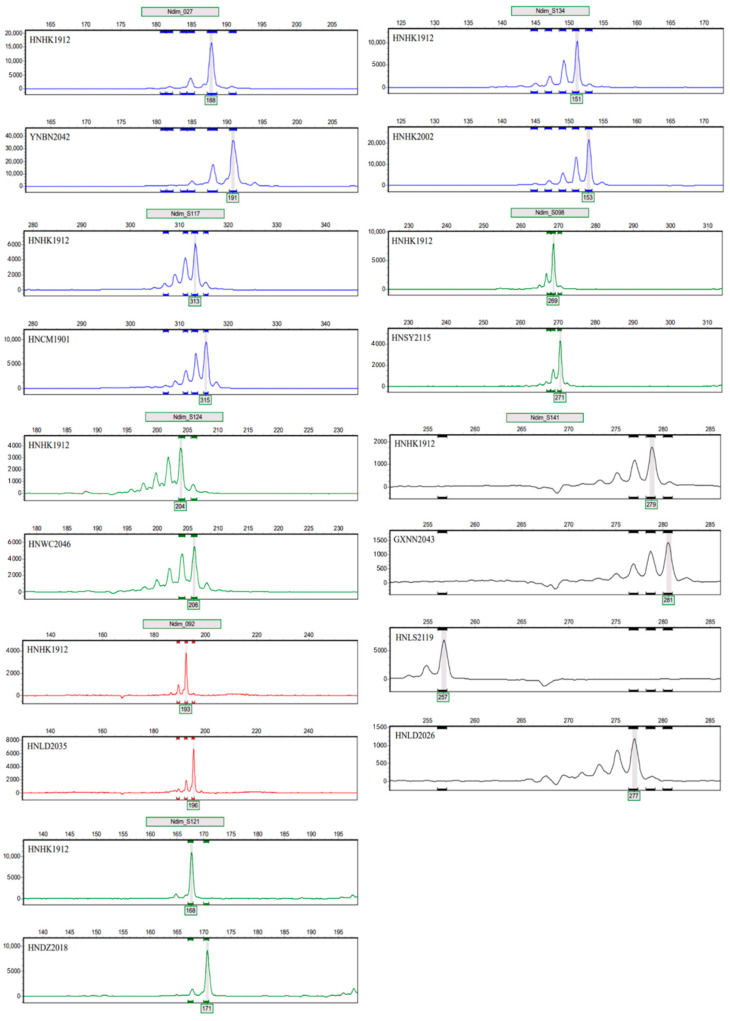
Peak plots of eight polymorphic microsatellite loci.

In order to accurately calculate the values of fluorescence signal peaks, four different fluorescent markers were added to the 5’ end of the primers (Table 4; Figure 5). The results showed that each microsatellite marker site has two or more alleles.

### 3.4. Validation of the Fluorescent Microsatellite Genotyping Assay

The power of the eight polymorphic microsatellite markers was evaluated with a panel of 35 *N. dimidiatum* isolates. The number of alleles (Na) ranged from two to four, and the polymorphism information content (PIC) values varied from 0.054 to 0.338, with a mean of 0.140 (Table 5).

**Figure 6:**
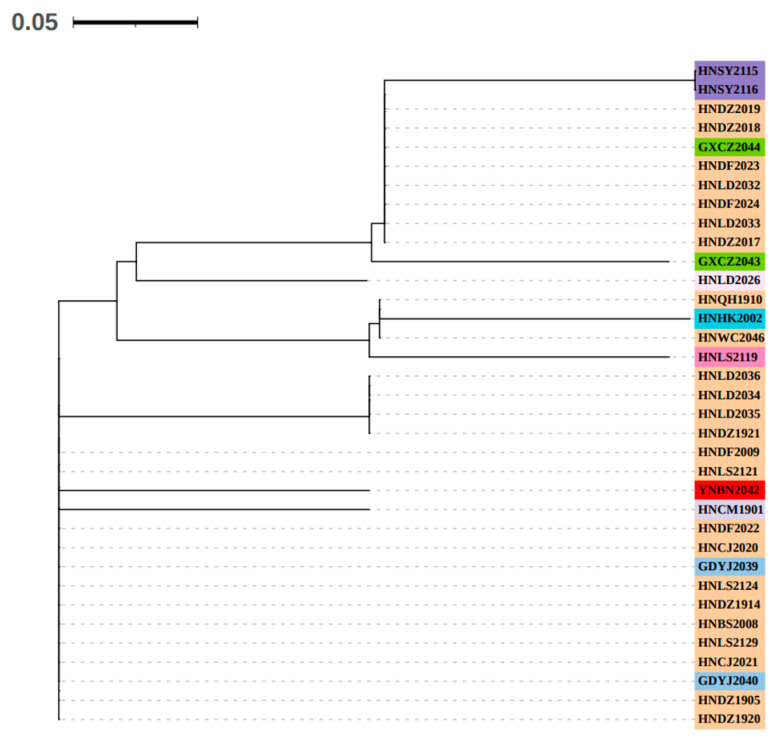
Neighbor-joining tree constructed based on the fluorescent microsatellite genotypes. Green, red and sky blue highlights represent the isolates collected from Guangxi autonomous region, Yunnan and Guangdong provinces of China, respectively. Medium purple, blush red, dark blue, orchid purple and lavender purple highlights represent the isolates collected from Hainan Province of China and independently branched. Details of the isolates used in this tree are shown in Table 1.

The neighbor-joining tree constructed with microsatellite genotypes indicated that there is a certain degree of genetic variation among the different isolates. The isolates collected from different cities or counties in the Hainan Province are scattered and distributed in different branches. Among them, two isolates collected from Sanya, Hainan (HNSY2115 and HNSY2116), are clustered into one branch; one isolate from Haikou, Hainan (HNHK2002), is branched separately; one isolate from Ledong, Hainan (HNLD2026), is branched separately; one isolate from Lingshui, Hainan (HNLS2119), is branched separately; and one isolate from Chengmai, Hainan (HNCM1901), is branched separately. The isolates collected from Chongzuo, Guangxi (GXCZ2043 and GXCZ2044); Yangjiang, Guangdong (GDYJ2039 and GDYJ2040); and Xishuangbanna, Yunnan (YNBN2042), were clustered with isolates from the Hainan Province (Figure 6).

## 4. Discussion

Pitayas are widely planted in tropical and subtropical regions around the world. The Hainan Province is an important pitaya production area in China. Cankers caused by *N. dimidiatum* represent a limiting factor for the development of the pitaya industry. The phylogenetic results of the present study show that our fungal isolates were grouped together with *N. hylocereum*, which has been proposed as a novel designated *Neoscytalidium* species that causes cankers on red-fleshed dragon fruit (*Hylocereus polyrhizus*) in Southern Thailand (Figure 1) [64]. However, the taxonomic relationships within *Neoscytalidium* species have been comprehensively reviewed recently. Following a comprehensive assessment, evidence strongly supports the synonymy between *N. hylocereum* and *N. oculus* with *N. dimidiatum* [7]. Hence, we still consider our fungal isolates as *N. dimidiatum* in the present study.

Genome-based molecular markers are important resources for the assessment of genetic diversity in pathogenic populations [65,66]. Therefore, it is of great significance to characterize the population dynamics of *N. dimidiatum* by means of genetic markers, so as to support the pitaya production chain. In the present study, the sequencing depth of the draft genome HNDZ1920 was 100×, and 11 GB of raw data was obtained. This assembled and annotated draft genome is able to meet the demand for microsatellite primer development. Additionally, the sequencing depth of whole-genome resequencing was 40×, and 5–6 GB of raw data for each sample was obtained, and their assembly processes were identical to the reference genome sequence HNDZ1920. The assembly qualities also met the demand for alignment with the draft genome. The base compositions of microsatellite loci on the draft genome are diverse, and the upstream and downstream sequences of most microsatellite loci are specific, thus implying that these candidate microsatellite loci have the potential to become widely used as genetic markers in population genetics and evolutionary studies. At the beginning of our study, we used the draft genome sequence only to screen the polymorphism microsatellite loci; however, only two loci with polymorphism between different isolates were screened, and the successful screening rate was approximately 1.02%, suggesting that this strategy has low screening efficiency. Our results are similar to those of previous reports in that the rate of discovering polymorphic microsatellite markers is still not high even if the development and screening of microsatellite markers are time-consuming and inefficient [67]. The authors of previous studies [68] have reported that combined resequencing strategies make it possible to improve the screening efficiency of microsatellite molecular markers. Hence, we further resequenced the genomes of another five *N. dimidiatum* isolates. Subsequently, we selectively designed 336 pairs of primers based on the resequencing comparison results to screen for microsatellite loci with polymorphisms and ultimately identified six polymorphic microsatellite loci. The successful screening rate was approximately 1.79%. Thus, screening efficiency increased by 75.49% compared to solely relying on the random screening of a single genome sequence.

We used a collection of 35 isolates with a wide sampling range to examine the power of microsatellite primers. As a measure of discriminatory power, the average PIC value of the developed markers in this study was 0.14 (<0.25), suggesting a low degree of discriminatory power according to the categorized criterion of the PIC values [69]. Both the low screening rate of microsatellites (1.79%) and the PIC range (0.054~0.338) of the eight pairs of polymorphic primers are most likely due to the limited degree of genetic variation among the isolates collected from different locations.

## 5. Conclusions

In the present study, we investigated the sequence differences in microsatellite loci, developed specific primers of the microsatellite polymorphism sites, and filled the gap in the presently available research tools for the characterization of the population structure and dynamics of *N. dimidiatum*. The eight pairs of microsatellite primers developed in the present study exhibited specificity and universality for the genotyping of *N. dimidiatum*, which have the potential to be widely used to genotype the pathogen worldwide. Their utilization will actively promote collaboration between laboratories around the world to conduct population research on *N. dimidiatum*, which is of great significance for the population variation and epidemic monitoring of this pathogen. Ultimately, however, there are still a large number of microsatellite loci in the genome sequence of *N. dimidiatum*, which is likely to have potential genetic variations among a screening panel of isolates with a wider genetic background than the panel used in the present study. Thus, it is still meaningful to continue to screen polymorphic microsatellite loci for the genotyping of the *N. dimidiatum* population, and in the future, more polymorphic microsatellite loci may be detected that were not discovered in our study.

## Figures and Tables

**Table 1:** Basic information on the *N. dimidiatum* used in the present study.

Isolates	Years	Locations	Reference Genome	Resequencing	Primer Screening
HNCM1901	2019	Chengmai, Hainan	-	-	Yes
HNDZ1905	2019	Danzhou, Hainan	-	-	-
HNQH1910	2019	Qionghai, Hainan	-	-	Yes
HNDZ1914	2019	Danzhou, Hainan	-	-	Yes
HNDZ1920	2019	Danzhou, Hainan	Yes	-	-
HNDZ1921	2019	Danzhou, Hainan	-	-	-
HNHK2002	2020	Haikou, Hainan	-	-	Yes
HNBS2008	2020	Baisha, Hainan	-	-	Yes
HNDF2009	2020	Dongfang, Hainan	-	-	-
HNDZ2017	2020	Danzhou, Hainan	-	-	-
HNDZ2018	2020	Danzhou, Hainan	-	-	-
HNDZ2019	2020	Danzhou, Hainan	-	-	-
HNCJ2020	2020	Changjiang, Hainan	-	-	Yes
HNCJ2021	2020	Changjiang, Hainan	-	-	-
HNDF2022	2020	Dongfang, Hainan	-	-	Yes
HNDF2023	2020	Dongfang, Hainan	-	-	-
HNDF2024	2020	Dongfang, Hainan	-	-	-
HNLD2026	2020	Ledong, Hainan	-	-	-
HNLD2032	2020	Ledong, Hainan	-	-	-
HNLD2033	2020	Ledong, Hainan	-	-	-
HNLD2034	2020	Ledong, Hainan	-	Yes	-
HNLD2035	2020	Ledong, Hainan	-	Yes	-
HNLD2036	2020	Ledong, Hainan	-	Yes	-
GDYJ2039	2020	Yangjiang, Hainan	-	Yes	Yes
GDYJ2040	2020	Yangjiang, Hainan	-	-	-
YNBN2042	2020	Xishuangbanna, Yunnan	-	-	Yes
GXCZ2043	2020	Chongzuo, Guangxi	-	-	Yes
GXCZ2044	2020	Chongzuo, Guangxi	-	-	-
HNWC2046	2020	Wenchang, Hainan	-	-	Yes
HNSY2115	2021	Sanya, Hainan	-	-	-
HNSY2116	2021	Sanya, Hainan	-	Yes	Yes
HNLS2119	2021	Lingshui, Hainan	-	-	-
HNLS2121	2021	Lingshui, Hainan	-	-	-
HNLS2124	2021	Lingshui, Hainan	-	-	Yes
HNLS2129	2021	Lingshui, Hainan	-	-	-

Note that all the isolates listed in this table were isolated from the pitayas and used for genotyping.

**Table 2:** Collection details and GenBank accession numbers of isolates used in the present study for phylogenetic analysis.

Species	Voucher/Culture	Location	GenBank Accession Number	
			ITS	*tub*
*N* *. dimidiatum*	Arp2-D	Turkey	MK813852	MK813852
*N. dimidiatum*	Kale4-C	Turkey	MK788362	MK788362
*N. dimidiatum*	Nd_Fig01	Turkey	OL304243	OK788660
*N. dimidiatum*	Nd_Fig02	Turkey	OL304244	OK788661
*N. dimidiatum*	Nd_Fig03	Turkey	OL304245	OK788662
*N. dimidiatum*	Ol_Dr04	Turkey	OK416080	OK428827
*N. dimidiatum*	Ol_Hl02	Turkey	OK416072	OK428819
*N. dimidiatum*	Ol_Nz19	Turkey	OK416079	OK428826
*N. dimidiatum*	Nd_Pp01	Turkey	OK643641	OK666382
*N. hyalinum*	CBS 125608	Gabon	MH863571	MT592752
*N. hyalinum*	CBS 125609	Gabon	MH863572	MT592753
*N. hyalinum*	CBS 125610	Gabon	MH863573	MT592754
*N. hyalinum*	CBS 125617	France	MH863577	MT592756
*Neoscytalidium* sp.	CBS 125619	France	MT587533	MT592757
*Neoscytalidium* sp.	CBS 125622	Martinique	MT587537	MT592765
*N. hyalinum*	CBS 125623	Martinique	MH863579	MT592766
*N. dimidiatum*	CBS 125695	France	KX464231	KX465065
*N. hyalinum*	CBS 125808	Martinique	MH863768	MT592767
*Neoscytalidium* sp.	CBS 137.77	USA	MT587535	MT592763
*S. hyalinum*	CBS 145.78	UK	KF531816	KF531796
*N. dimidiatum*	CBS 251.49	USA	KF531819	KF531799
*Neoscytalidium* sp.	CBS 312.90	Netherlands	MT587536	MT592764
*N. dimidiatum*	CBS 499.66	Mali	KF531820	KF531800
*Neoscytalidium* sp.	CBS 601.85	USA	MT587538	MT592768
*Neoscytalidium* sp.	CBS 662.77	-	MT587534	MT592758
*N. hyalinum*	CMM 3566	Brazil	KF234551	KF254935
*N. hyalinum*	CMM 3649	Brazil	KF234550	KF254934
*N. hyalinum*	COUFAL 0144	Brazil	MH251953	MH251969
*N. hyalinum*	COUFAL 0145	Brazil	MH251954	MH251970
*N. hyalinum*	COUFAL 0146	Brazil	MH251955	MH251971
*N. dimidiatum*	DE 1606	China	KY013660	KY349087
*N. hylocereum*	PSU-HP01	Thailand	LC590859	LC647832
*N. hylocereum*	TSU-HP01	Thailand	LC590860	LC647833
*N. hylocereum*	TSU-HP02	Thailand	LC590861	LC647834
*N. novaehollandiae*	CBS 122070	Australia	EF585539	MT592759
*N. novaehollandiae*	CBS 122071	Australia	EF585540	MT592760
*N. novaehollandiae*	CBS 122072	Australia	EF585535	MT592761
*N. novaehollandiae*	CBS 122610	Australia	EF585536	MT592762
*Neoscytalidium* sp.	IOM 325287	Mexico	MG764431	-
*N. orchidacearum*	MFLUCC 12-0533	Thailand	KU179865	-
*N. dimidiatum*	HNDZ1920	China	PP917774	PP928423
*N. dimidiatum*	HNLD2034	China	PP917775	PP928424
*N. dimidiatum*	HNLD2035	China	PP917776	PP928425
*N. dimidiatum*	HNLD2036	China	PP917777	PP928426
*N. dimidiatum*	GDYJ2039	China	PP917778	PP928427
*N. dimidiatum*	HNSY2116	China	PP917779	PP928428
*B. dothidea* (Outgroup)	CMW 8000	Switzerland	AY236949	AY236927

**Table 3:** Genome assembly results of *N. dimidiatum*.

Genome Features	*N. dimidiatum* Genome
Number of contigs	578
Assembled genome size (bp)	43,460,451
Contig N50 (bp)	266,892
Contig L50	45
Largest contig	1,170,203
GC content of the genome (%)	53.96
Ns per 100 kbp	3.41
Content of repeat sequences (%)	2,801,256 bp (6.45%)
tRNA	138
rRNA	15
sRNA	3
snRNA	38
Predicted protein-coding genes (#)	9863

**Table 4:** Polymorphic SSR site-specific primers of *N. dimidiatum*.

Locus	Primer	Product Length (bp)	Motif	Fluorophore
Ndim027	F:5′-GAGCAAAGGACACCAAAGCG-3′	188–191	AAG	FAM
	R:5′-GTCTCGATCTTGGTCGTCGG-3′			
Ndim092	F:5′-TCGCACAACACTTCGCAAAG-3′	193–196	AAG	ROX
	R:5′-TGGATCGACGCCTTTGGAAA-3′			
NdimS098	F:5′-TTGGGTCCAGCTTGTGTTGT-3′	269–271	GA	HEX
	R:5′-GGTGCCTGCTCATTACGGTA-3′			
NdimS117	F:5′-CTCTTTGTCCGCTGGATGGT-3′	313–315	CT	FAM
	R:5′-ACAAGCCCCATACCCGTAAC-3′			
NdimS121	F:5′-CATCGAACGCATGCAAGAGG-3′	168–171	ACC	HEX
	R:5′-GGAGAAAGGCGTGCTCATGT-3′			
NdimS124	F:5′-ACACCTTTCTAGCGCAGTCC-3′	204–206	AG	HEX
	R:5′-TGAAGGTCTGGTCGATGTGC-3′			
NdimS134	F:5′-ATGTCGGCGCGTTATCTGAT-3′	151–153	GT	FAM
	R:5′-GGGTCCAGAATTCTCACCGG-3′			
NdimS141	F:5′-TCAAACGCTTCCCCTTCCTC-3′	257–281	CT	TAMRA
	R:5′-TGAGGAAGGAATCGATCGCG-3′			

Locus, microsatellite molecular markers; primer, F: forward primer and R: reverse primer; product length (bp), PCR products’ length; motif, SSR repeat motif; fluorophore, primer modification: FAM, ROX, HEX, and TAMRA.

**Table 5:** Polymorphism information content of 8 SSRs in 35 *N. dimidiatum* isolates.

Locus	k	N	PIC
Ndim027	2	35	0.054
Ndim092	2	35	0.182
NdimS098	2	35	0.102
NdimS117	2	35	0.054
NdimS121	2	35	0.338
NdimS124	2	35	0.182
NdimS134	2	35	0.054
NdimS141	4	35	0.158

Locus, microsatellite molecular markers; k, number of alleles; N, number of isolates; PIC, polymorphism information content.

**Figure A9:**
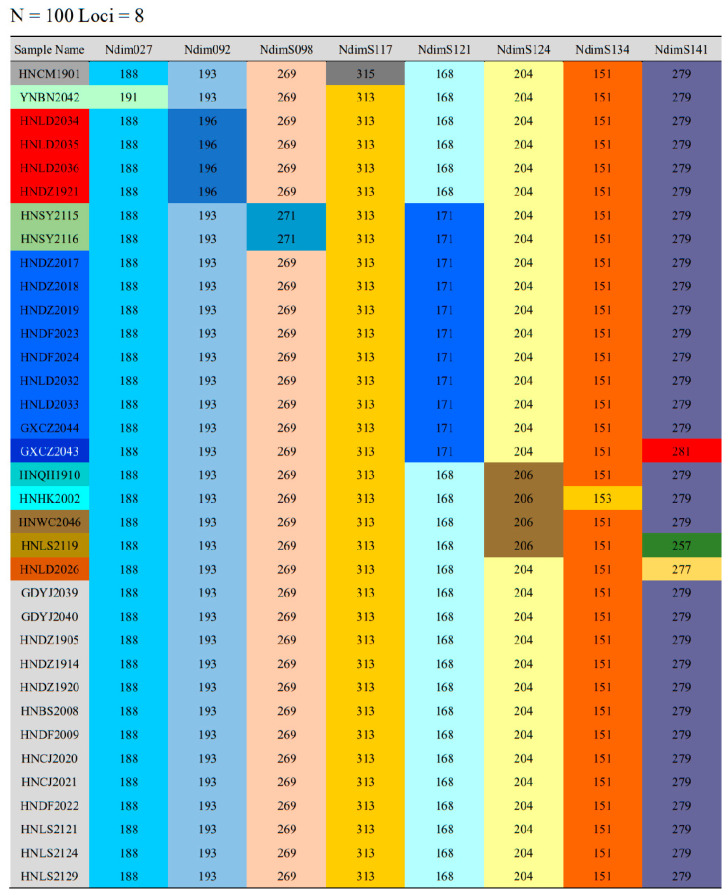
Fingerprint of 35 *N. dimidiatum* isolates. N, the total number of the samples; loci, the total number of the microsatellite markers.

## Data Availability

The genome sequence and resequencing assembly results of *N. dimidiatum* isolates are available in NCBI under BioProject: PRJNA981440, BioSample: SAMN35719075, SAMN41487665, SAMN41487666, SAMN41487667, SAMN41487668, and SAMN41487669.
